# Cardiology-Chat: A Multi-LLMs Powered System for Cardiac Diagnostic Reasoning and Clinical Support

**DOI:** 10.1109/JTEHM.2026.3668755

**Published:** 2026-02-26

**Authors:** Zhibin Yang, Chuanyue Chen, Seedahmed S. Mahmoud, Xuerui Tan, Yequn Chen, Qiang Fang

**Affiliations:** Department of Biomedical EngineeringCollege of EngineeringShantou University 12386 Shantou Guangdong 515063 China; The First Affiliated Hospital of Shantou University Medical College Shantou Guangdong 515041 China

**Keywords:** Cardiology medical diagnosis, clinical decision support, large language models (LLMs), retrieval-augmented generation, supervised fine-tuning

## Abstract

Cardiovascular diseases are a leading global cause of death, but their accurate diagnosis remains challenging. While Large Language Models (LLMs) show promise in assisting disease diagnosis in general, their adoption in cardiology is hindered by three critical limitations: hallucination, inadequate domain-specific reasoning, and restricted knowledge coverage. To overcome these barriers, we developed Cardiology-Chat, an LLM-based system specifically tailored for cardiology. The system employs a three-step main reasoning framework: 1) parsing user queries with Llama 3.1 8B-instruct to extract key clinical information; 2) retrieving evidence from the knowledge base via Retrieval-augmented generation (RAG); and 3) generating diagnostic conclusions using the fine-tuned Llama model. Two critical components have been developed to support the system’s functionality. The first is a specialized cardiovascular vector knowledge base, constructed from multiple data sources to enhance the RAG subsystem. The second is a Chain-of-Thought–augmented dataset designed to strengthen the LLM’s in-depth reasoning capabilities. In addition, multiple LLMs were adopted to mitigate the possible “self-consistency” bias. Experiments on public cardiology QA and real clinical cases demonstrated significant performance improvements, achieving 0.796 accuracy and 0.807 F1 respectively.

## I. Introduction

Cardiovascular diseases (CVD) are the leading cause of death worldwide. According to the World Health Organization, approximately 18 million people die annually from CVD, representing 32% of all deaths [1]; however, their accurate and timely diagnosis presents a persistent challenge, often requiring the complex integration of heterogeneous patient data and deep domain expertise [2]. The emergence of large language models (LLMs) such as ChatGPT, Gemini, and DeepSeek has revolutionized natural language processing [3] and demonstrated compelling potential in the broader medical field for handling complex textual information, including patient records and clinical notes [4]. However, directly applying general-purpose LLMs to highly specialized medical domains such as cardiology faces significant hurdles [5]. These models lack the requisite depth of expertise and sophisticated clinical reasoning abilities [6]. Specifically, three key issues exist. At first, due to the absence of specialized knowledge constraints, LLMs are prone to “hallucinations,” generating seemingly plausible but clinically inaccurate or even harmful medical advice [7]. Secondly, when confronted with complex cardiology cases, existing models exhibit limited multi-step reasoning and differential diagnosis capabilities, struggling to emulate the diagnostic thought processes of specialist physicians [8]. Finally, even LLMs fine-tuned on general medical data suffer from incomplete knowledge coverage, particularly regarding emerging treatment options and rare conditions [9].

Retrieval-augmented generation (RAG) frameworks have emerged as a promising approach to mitigate hallucinations and incorporate external knowledge [10]. However, standard RAG implementations face specific challenges in cardiology. The complexity of cardiac cases often necessitates synthesizing information dispersed across multiple documents or different data types that change over time [11]. Traditional RAG, typically retrieving isolated text chunks, struggles to address this information fragmentation, potentially leading to incomplete or biased diagnostic insights [12]. Furthermore, the quality, timeliness, and domain-specific relevance of the underlying knowledge base are crucial but often lacking in general-purpose medical RAG systems [13]. Similarly, while supervised fine-tuning (SFT) can enhance model capabilities, it often relies on static training data, unable to keep pace with the dynamic nature of medical knowledge, and frequently fails to explicitly train the complex, multi-step reasoning required in clinical practice [14]. To address this limitation, we propose a retrieval-augmented generation (RAG) architecture that allows the model to leverage both its internal knowledge and up-to-date medical information during inference.

Addressing these challenges, we developed Cardiology-Chat, an multiple-LLM based assistive system specifically designed for medical diagnosis in cardiology. This system innovatively integrates:

1) A Structured Three-Step Reasoning Framework: Guiding the diagnostic process from initial query understanding and information extraction, through evidence retrieval via RAG, to final complex reasoning by a fine-tuned LLM.
2) A Cardiology-Specific Knowledge Base: We constructed a cardiology vector knowledge base, integrating authoritative medical guidelines, expert consensus, and the latest research findings, to provide a reliable and retrievable source of external knowledge for RAG.
3) A Cardiology Reasoning Model: We enhanced the model’s reasoning capabilities to handle complex clinical cases through Chain-of-Thought (CoT) data generation, using the generated data as a training set for model fine-tuning.
4) A Task-Optimized Multi-LLM Architecture: We integrate multiple LLMs: Llama 3.1 8B-instruct forms the basis for our fine-tuned reasoning model, while the use of distinct DeepSeek models (V3 for generation, R1 for validation) are crucial for generating high-fidelity CoT data and ensuring robustness against bias, enhancing the overall effectiveness and efficiency.

## II. Related Work

### A. AI Evolution in Cardiology

AI in cardiovascular medicine has evolved from traditional statistical risk scores to advanced machine learning and deep learning algorithms. Machine learning has been widely applied to structured EHR data for tasks like heart failure prediction [15]. More recently, deep learning analyzes specific modalities such as cardiovascular imaging, showing potential in tasks like view classification and segmentation in cardiac ultrasound [16]. However, these methods struggle to integrate diverse, unstructured clinical data. Large language models (LLMs) offer transformative potential in this regard. The emergence of LLMs offers transformative potential. Examples include LLMs like ChatGPT being used to automatically generate cardiac ultrasound reports, significantly improving diagnostic accuracy and efficiency [17], thus further advancing AI application in cardiovascular medicine.

### B. LLM Limitations in Cardiology

Although general medical LLMs such as Med-PaLM 2 and GPT-4 demonstrate broad capabilities, their direct application in specialized areas like cardiology exposes significant limitations [18]. Attempts to specifically adapt LLMs to cardiology, such as ZODIAC [19] through domain-specific fine-tuning, have validated potential but also highlighted persistent gaps. Major shortcomings include a lack of robust grounding to external evidence and insufficient clinical case reasoning capabilities [20]. These limitations highlight the need to move beyond traditional fine-tuning methods.

### C. Limitations of RAG for Cardiology

RAG aims to mitigate knowledge limitations by integrating external sources. Although it has been explored in various biomedical contexts, standard RAG faces hurdles in supporting the complex clinical reasoning required in cardiology. For instance, explorations include using RAG for zero-shot ECG diagnosis, highlighting its potential in bridging the gap between textual tasks and cardiac disease diagnosis [21]; developing customized chatbots for cardiology inquiries aimed at reducing hallucinations and improving performance [22]; and proposing a method for ECG report generation and question answering via retrieval-augmented self-supervised modeling, showcasing its effectiveness in patient care improvement [23]. Despite these efforts, many RAG systems still lack the domain-specific depth and ability to synthesize fragmented evidence crucial for complex cardiac diagnoses.

### D. CoT for Clinical Reasoning

CoT prompting improves LLM reasoning by eliciting intermediate steps [24], [25]. While beneficial in medical question answering, obtaining expert-annotated CoT is costly [26]. With ongoing advancements, methods for automatically generating more sophisticated CoT data have emerged, including approaches like those explored in HuatuoGPT-o1, often involving iterative refinement and validation [27]. However, a key challenge remains in grounding generated CoT data in reliable, evidence-based reasoning, especially in specialized domains like cardiology where domain expertise is crucial.

## III. Method

### A. The Architecture of Cardiology-Chat

**FIGURE 1.**
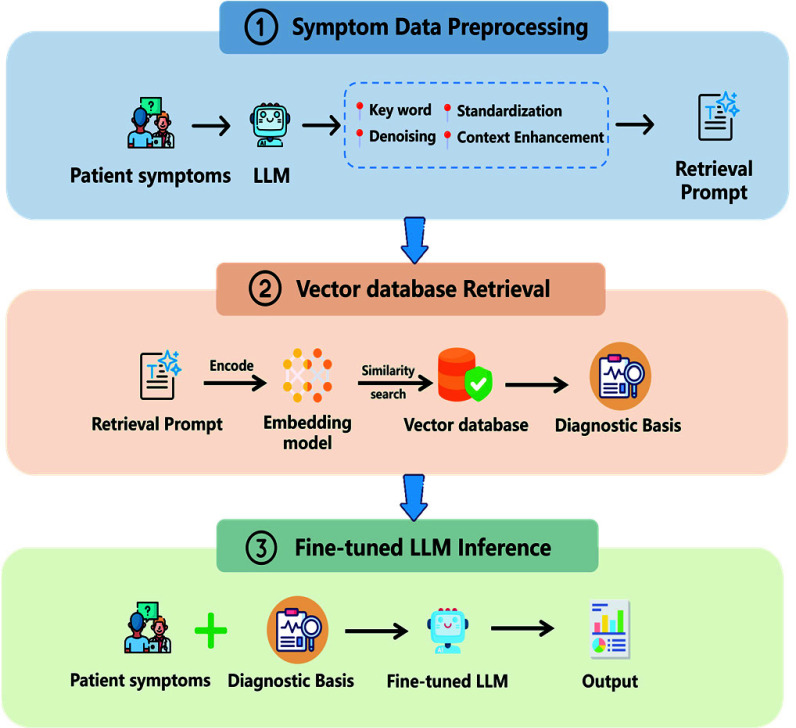
Cardiology-Chat System Workflow.

The proposed system employs a structured, multi-stage processing architecture with a three-step main reasoning framework (shown in Fig. 1).

As shown in Fig. 1, the three steps of the system are as follows:

Stage 1: Symptom Data Preprocessing. This stage aims to transform the user’s raw natural language input (containing symptoms, signs, or related inquiries) into a structured format suitable for efficient retrieval. This process utilizes Llama 3.1 8B-instruct to process the input text. Llama 3.1 8B-instruct was selected for its demonstrated ability to effectively process natural language and follow instructions [28], providing sufficient capabilities for extracting and structuring key clinical information relevant to the subsequent retrieval stage. Key steps include extracting core medical keywords and relevant attributes (e.g., symptoms, location, duration), removing redundant or irrelevant noise, standardizing diverse natural language expressions into uniform medical terminology, and structuring the extracted information to enhance contextual association. The goal of this stage is to reduce ambiguity in natural language, unify clinical terminology, and ultimately generate a refined, structured “Retrieval Prompt” to serve as a direct input for knowledge retrieval in the next stage, enabling more precise and efficient similarity matching.
Stage 2: Vector Database Retrieval. This stage employs the core mechanism of RAG to acquire relevant evidence-based medical knowledge. The system utilizes the “Retrieval Prompt” generated in the previous stage to perform a semantic similarity search within a specifically constructed cardiovascular disease vector knowledge base. This process retrieves knowledge snippets most relevant to the user’s symptoms. These snippets are primarily sourced from authoritative clinical guidelines and reliable medical information sources, collectively forming the “Diagnostic Basis,” which provides a solid foundation of external knowledge and factual evidence to support the final inference process.
Stage 3: Fine-tuned LLM Inference. This is the core intelligent analysis and diagnostic decision-making stage of the system. In this stage, the “Diagnostic Basis” retrieved in the second stage, along with the patient’s original symptom description, are provided as input to the specifically fine-tuned Cardiology-Chat model. The model leverages its complex clinical reasoning abilities acquired through CoT training to deeply integrate and analyze the input symptom information and retrieved evidence-based knowledge, ultimately outputting concrete diagnostic results.

### B. Data Sources

**FIGURE 2.**
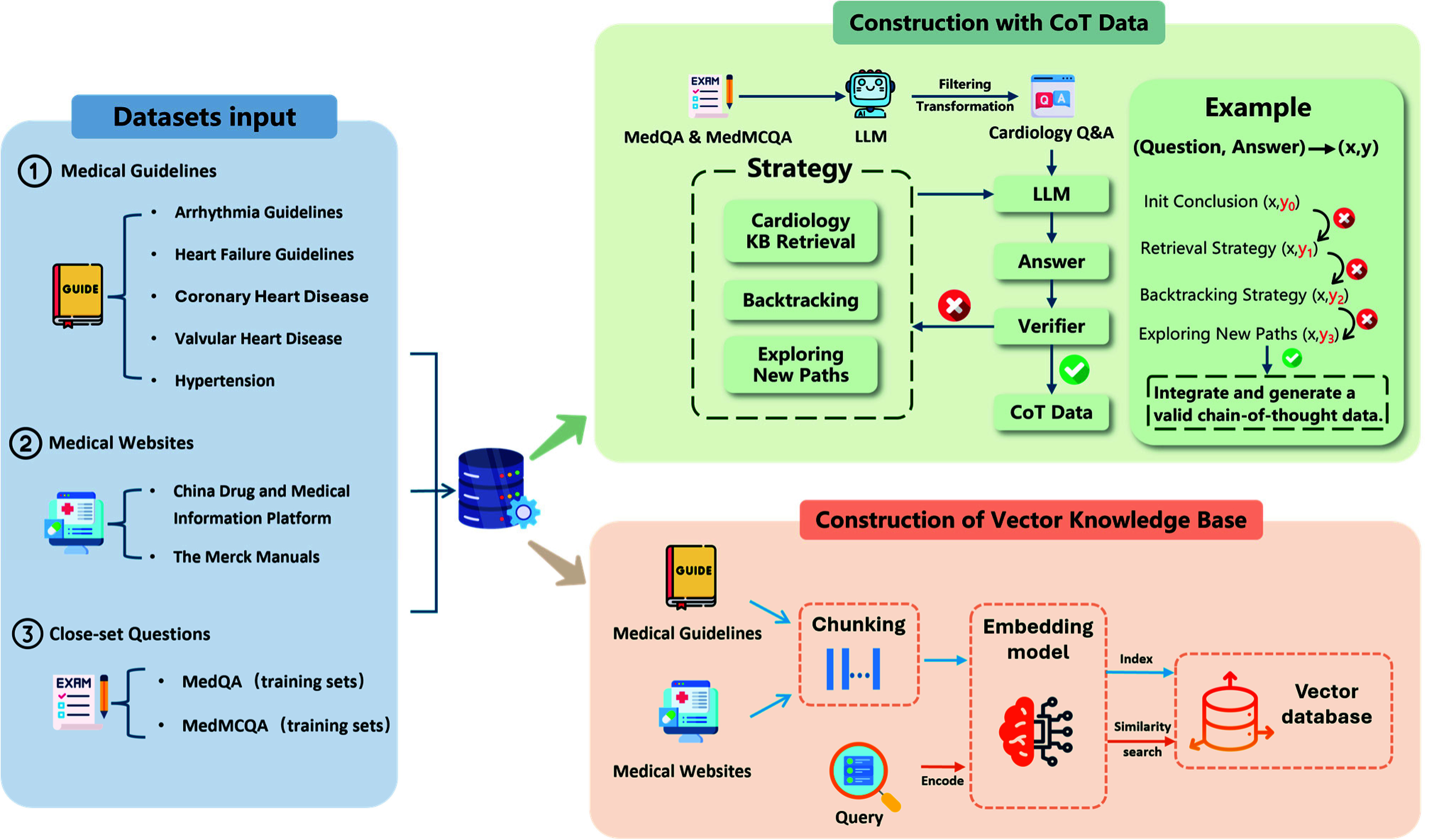
Cardiology-Chat Knowledge Base and CoT Training Dataset Construction Process.

This section focuses on constructing two key datasets that underpin the system’s functionality: a specialized cardiovascular vector knowledge base supporting the RAG framework; and a CoT augmented dataset for cultivating the large language model’s in-depth reasoning abilities, as illustrated in Fig. 2.

### 2) Construction of Cardiology-Specific Vector Knowledge Base

To establish a reliable knowledge source for the RAG component, the unstructured text from guidelines and websites was systematically processed. First, DeepSeek-V3 [31] was used to convert the source documents into a hierarchical Markdown format that preserved the structure of headings, lists, etc. Then, semantic chunking was performed based on the heading structure, treating each section and its content as an independent unit to maintain semantic integrity. Subsequently, the text-embedding-3-large model was used to transform the text blocks into high-dimensional vectors, capturing deep semantic information, ultimately constructing the vector knowledge base.

### 3) Generation of Chain-of-Thought Training Data

This study constructs the cardiovascular CoT dataset through an iterative generation-verification-correction loop (Fig. 2, upper right). Given cardiovascular question–answer pairs (x,y), DeepSeek-V3 first acts as a generator, progressively producing a candidate reasoning path and a provisional answer y0. Notably, y0, is not presumed correct; it must be validated by DeepSeek-R1 [32], acting as an independent validator.

Using distinct DeepSeek releases (V3 for generation, R1 for validation) is critical for reliability and objectivity. The model diversity mitigates self-consistency bias—where a generator could tacitly accept its own reasoning—and yields a more stringent, external-like review of CoT quality.

Verification of generator outputs. R1 applies two complementary checks to each generated CoT:

1) Answer agreement, testing whether the provisional answer y_0_ matches the reference y; and
2) Reasoning-quality auditing. In this second check, R1 re-analyzes the entire CoT path and cross-checks intermediate statements against structured cardiovascular knowledge retrieved from the vector knowledge base (Fig. 2) to assess factual alignment with authoritative guidelines and step-wise logical coherence.

A reasoning chain is accepted only if both checks pass—that is, the final answer agrees and the reasoning is guideline-aligned and internally coherent. Even when y ${}_{0} =$ y, the chain is rejected if it fails the reasoning-quality auditing.

Failure handling. If validation fails, the pipeline triggers one of three repair strategies (chosen at random without replacement), followed by re-validation:

Knowledge Base Retrieval: Retrieve relevant knowledge fragments from the vector knowledge base based on the question x and intermediate steps to assist in generating a new answer y1.

Retrospective Correction: Reanalyze and generate y2 for the problem steps pointed out by the validator.

Path Reconstruction: Abandon the original reasoning path and attempt a completely new solution to generate y3.

If the chosen strategy still fails, another strategy is attempted; after all three have been tried, we restart the full process for that item, allowing up to three end-to-end retries. Items that still fail are discarded to ensure data quality.

Across all generation rounds, approximately 7k CoT instances (out of 9.2k candidates) passed both checks, yielding a 76% acceptance rate. Each accepted item was stored as a high-quality annotated triplet (x, CoT_path, y ${}_{\mathrm {final}}$). The custom-generated questions were sourced from MedQA and MedMCQA, and the resulting 7k verified CoTs were combined with 20k publicly available CoT examples to form the final 27k dataset for supervised fine-tuning.

To provide a direct baseline for the ablation study, we also created a simple CoT dataset using the same generator (DeepSeek-V3) but without any validation or correction. Each cardiovascular question from MedQA and MedMCQA was prompted once to produce a single reasoning path and final answer, with no involvement of R1 or knowledge retrieval. This baseline allows clear isolation of the contribution of the verification and repair stages.

All custom CoT samples were generated exclusively from the training splits (MedQA-train, MedMCQA-train), while evaluation relied on non-overlapping validation and test sets (MedQA-test, MedMCQA-val, PubMedQA-test). This strict data separation prevents leakage and ensures the integrity of the fine-tuning and evaluation pipelines.

## C. Model Training and Fine-Tuning

For the full-parameter supervised fine-tuning, the Llama 3.1 8B-instruct model (from Meta AI) was selected as the foundation large model. This choice was made considering its general language modeling capabilities and suitability as a base model for training on specialized domain data, along with its balanced performance profile. The compiled 27k CoT dataset was then utilized for this fine-tuning process. Data preprocessing used a maximum sequence length of 8192 tokens and instruction alignment was performed using a template format compatible with LLaMA 3 Instruct. Training adopted a standard language model autoregressive loss function, with hyperparameters were set as follows: training for 3 epochs; initial learning rate of 5e-6, using the AdamW optimizer (weight decay coefficient of 0.1, weight decay not applied to bias terms and LayerNorm parameters); using a cosine decay learning rate scheduler, with a warm-up ratio of 5% of the total training steps; the training was conducted on an in-house server equipped with $2\times $ NVIDIA A800 GPUs. Each GPU used a batch size of 6, and gradient accumulation was performed for 16 steps across 2 GPUs, resulting in an effective batch size of 192. Gradient checkpointing and BF16 mixed-precision training were also enabled. Compared to the base model, the fine-tuned Cardiology-Chat 8B model demonstrates significantly improved query understanding capabilities and evidence-based reasoning levels on cardiovascular-specific datasets.

## IV. Experiments

### A. Evaluation Datasets and Benchmarks

**FIGURE 3.**
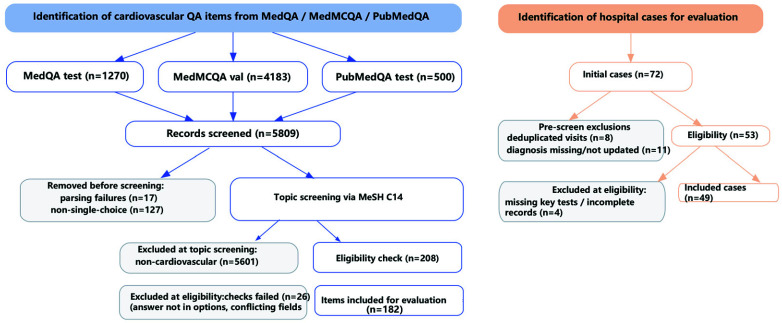
Data screening and inclusion workflow for evaluation datasets.

Standard Medical Benchmarks (QA Task): To standardize the evaluation of the model’s fundamental knowledge mastery and reasoning ability, we constructed a cardiology-specific multiple-choice question dataset. This dataset was derived from three authoritative public medical question-answering benchmarks: MedQA (test set portion based on USMLE practice questions), MedMCQA (validation set portion), and PubMedQA (test set portion) [33]. Cardiology QA screening pipeline. We constructed the test set via a three-stage, pipeline (Fig. 3). Starting from MedQA-test (n=1,270), MedMCQA-val (n=4,183), and PubMedQA-test (n=500) (total n=5,809), we applied: (i) pre-screening (removed parsing failures n=17 and non-single-choice items n=127); (ii) topic screening via MeSH C14, where entities were extracted by an LLM and mapped to MeSH to exclude non-cardiovascular items (n=5,601 excluded); and (iii) eligibility checks (answer present in options; no conflicting fields), removing n=26. The final cardiology test set contains 182 questions distributed across four subcategories, i.e., Structural (52), Functional (54), Coronary & Vascular (46), and Special (30).

In-house real clinical dataset (Diagnostic List Generation Task): To evaluate the model’s practicality and extensibility in real-world clinical scenarios, we used a dataset of 49 anonymized cardiology cases from the First Affiliated Hospital of Shantou University Medical College (IRB approval No. B-2025-065, ethics date of approval: May 26, 2025; all personally identifiable information was removed prior to analysis). Each case contains the patient’s clinical summary (chief complaint, history, physical exam, ancillary tests) and a corresponding ground-truth diagnosis list established by the hospital’s senior cardiologists. This expert-verified diagnosis list serves as the reference standard for evaluating the model’s performance. The dataset is used solely for testing, with Precision, Recall, and F1 score as the evaluation metrics. The inclusion flow is provided in Fig. 3 (initial n=72; pre-screen exclusions: deduplicated visits n=8; missing/not-updated diagnosis n=11; eligible n=53; excluded at eligibility n=4; included n=49).

### B. Evaluation Metrics

Cardiology-Chat was evaluated using quantitative, qualitative, and system level metrics, organized by target as follows.

#### 1) Benchmark QA Evaluation

On MedQA, MedMCQA, and PubMedQA, Accuracy is reported as the primary correctness measure. The cross target metrics Calibration and Top k coverage are also included to assess confidence alignment and robustness under ranked choice settings.

#### 2) Real-Clinical Evaluation (Diagnosis List Generation)

**FIGURE 4.**
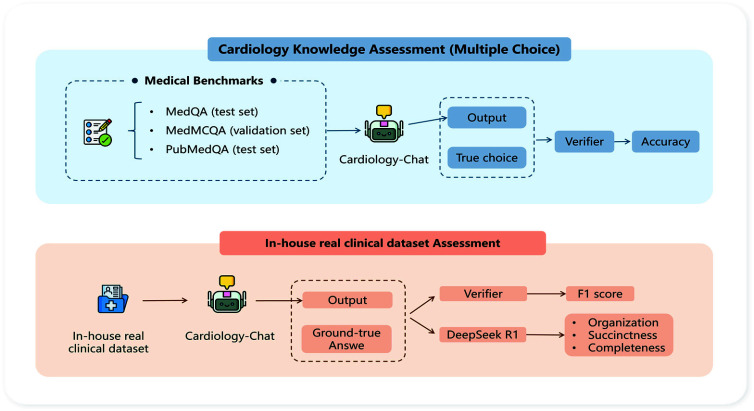
Evaluation workflow for Cardiology-Chat.

For 49 in house cases, Precision, Recall, and F1 are computed between model generated and expert diagnosis lists (Fig. 4). The same cross target metrics, Calibration and Top k coverage, are adapted to ranked diagnosis lists. Moreover, a deterministic LLM assessor (DeepSeek R1; T = 0; fixed prompt) rates Organization, Completeness, and Succinctness on a scale from 1 to 100.

#### 3) System-Level Efficiency Evaluation

p50 and p95 per sample and per batch latency, time to first token (TTFT), and throughput in tokens per second are reported. For the RAG component, retriever quality is measured by Support@k, Relevant Hit@k, and Context Precision@k to quantify evidence contribution.

### C. Evaluation Results

We compared Cardiology-Chat 8B with five baselines: three general LLMs (Qwen-2.5-7B-Instruct, LlaMA 3.1 8B-Instruct, DeepSeek-R1-Distill-Llama-8B) and two medical-domain LLMs (BioMistral-7B, HuatuoGPT-o1-7B). We also include an ablated variant of our model without retrieval (Cardiology-Chat 8B w/o RAG).

#### 1) Benchmark QA Evaluation

Cardiology-Chat 8B leads the cardiology QA benchmark (n=182) with 0.796 average accuracy across four subcategories (Table 1), surpassing Llama 3.1 8B-Instruct (0.612, + 18.4 pp) and the no-RAG ablation (0.703, + 9.3 pp). Category gains are consistent, strongest in Structural (+ 22.4 pp) and Functional (+ 21.0 pp), underscoring the benefit of structured retrieval. Ranked-choice performance is higher as well: Top-1/3/ $6=41.8$/69.8/76.9% (±95% CI; Table 3) vs 36.4/61.2/72.5% without RAG, indicating better confidence alignment.

**TABLE 1:** Performance Comparison (Accuracy) of Cardiology-Chat and Baseline Models on the Cardiology QA Benchmark Dataset

Model	Structural Heart Disease	Functional Heart Disease	Coronary and Vascular Disease	Special HeartDiseases	Avg.
Qwen2.5-7B-Instruct	0.461	0.672	0.601	0.522	0.564
LLaMA-3.1-8B-Instruct	0.583	0.642	0.579	0.644	0.612
BioMistral-7B	0.473	0.591	0.654	0.496	0.553
HuatuoGPT-o1-7B	0.683	0.732	0.621	0.640	0.669
DeepSeek-R1-Distill-Llama-8B	0.461	0.476	0.557	0.466	0.490
Cardiology-Chat 8B	0.807	0.851	0.760	0.766	0.796
Cardiology-Chat 8B w/o RAG	0.711	0.773	0.633	0.696	0.703

**TABLE 2:** Performance Comparison of Cardiology-Chat and Baseline Models on the In-House Dataset

Model	F1-score			LLM Qualitative Metrics		
	Precision	Recall	F1	Organization	Completeness	Succinctness
Qwen2.5-7B-Instruct	0.612	0.564	0.587	0.642	0.683	0.809
LLaMA-3.1-8B-Instruct	0.622	0.528	0.571	0.663	0.621	0.812
BioMistral-7B	0.562	0.591	0.576	0.541	0.589	0.721
HuatuoGPT-o1-7B	0.722	0.611	0.662	0.624	0.617	0.823
DeepSeek-R1-Distill-Llama-8B	0.654	0.595	0.623	0.671	0.668	0.783
Cardiology-Chat 8B	0.853	0.766	0.807	0.827	0.874	0.787
Cardiology-Chat 8Bw/o RAG	0.792	0.671	0.726	0.811	0.816	0.839

**TABLE 3:** Top-K Accuracy (TOP-1/3/6) With 95% Confidence Intervals

Domain	Top 1(Accuracy and 95% CI)		Top 3(Accuracy and 95% CI)		Top 6 (Accuracy and 95% CI)	
	QA	In-house	QA	In-house	QA	In-house
Qwen2.5-7B-Instruct	27.8% [0.21, 0.34]	27.1% [0.21, 0.34]	45.2% [0.37, 0.53]	44.6% [0.36, 0.53]	58.6% [0.50, 0.66]	57.9% [0.50, 0.65]
LLaMA-3.1-8B-Instruct	28.6% [0.22, 0.35]	27.9% [0.21, 0.35]	46.1% [0.38, 0.54]	45.3% [0.36, 0.54]	59.4% [0.51, 0.67]	58.1% [0.50, 0.66]
BioMistral-7B	23.6% [0.16, 0.28]	22.1% [0.15, 0.27]	37.4% [0.29, 0.46]	35.5% [0.28, 0.44]	49.7% [0.42, 0.55]	46.1% [0.37, 0.51]
HuatuoGPT-o1-7B	30.2% [0.24, 0.37]	30.9% [0.24, 0.37]	49.5% [0.42, 0.57]	49.1% [0.44, 0.56]	62.1% [0.55, 0.69]	61.8% [0.53, 0.66]
R1-Distill-Llama-8B	22.3% [0.16, 0.29]	20.8% [0.13, 0.25]	34.8% [0.23, 0.49]	30.1% [0.22, 0.43]	48.2% [0.41, 0.55]	44.5% [0.39, 0.51]
Cardiology-Chat 8B	41.8% [0.35, 0.49]	40.1% [0.32, 0.44]	69.8% [0.63, 0.76]	66.3% [0.60, 0.72]	76.9% [0.70, 0.82]	72.1% [0.66, 0.79]
w/o RAG	36.4% [0.30, 0.43]	34.7% [0.28, 0.41]	61.2% [0.54, 0.68]	58.9% [0.51, 0.66]	72.5% [0.65, 0.79]	68.3% [0.60, 0.75]

#### 2) Real-Clinical Evaluation (Diagnosis List Generation)

On 49 in-house cases, Cardiology-Chat 8B achieves the best diagnosis-list scores (Table 2): Precision/Recall/F $1=0.853$/0.766/0.807, improving over the no-RAG variant (0.792/0.671/0.726) by + 0.061/+ 0.095/+ 0.081. Cross-target metrics on this clinical set also improve: Top-1/3/6 = 40.1/66.3/72.1% vs 34.7/58.9/68.3% without RAG (95% CIs in Table 3), and Brier/ECE = 0.598/0.589 vs 0.685/0.671 without RAG (Table 5). A deterministic rubric (DeepSeek-R1, T=0) further rates Organization 0.827 and Completeness 0.874, with a small drop in Succinctness 0.787 (vs 0.839 w/o RAG), indicating richer evidence-grounded outputs with slight redundancy.

**TABLE 4:** System-Level Efficiency Metrics (Latency, Time-to-First-Token, and Throughput) of Cardiology-Chat and Baseline Models)

Model	p50 (ms)		p95 (ms)		TTFT p50 (ms)		Throughput (tokens/s)	
	QA	In-house	QA	In-house	QA	In-house	QA	In-house
Qwen2.5-7B-Instruct	84.63	96.42	145.02	166.87	25.91	31.33	4 680.12	4 512.09
LLaMA-3.1-8B-Instruct	97.55	109.73	159.18	181.42	28.46	33.27	4 230.47	4 080.53
BioMistral-7B	82.18	93.74	142.05	161.09	25.38	30.25	4 732.64	4 590.12
HuatuoGPT-o1-7B	88.70	99.55	150.12	171.68	26.97	31.90	4 540.23	4 365.77
DeepSeek-R1-Distill-Llama-8B	99.32	111.08	162.76	184.24	29.22	34.10	4 155.84	3 996.31
Cardiology-Chat 8B	105.50	118.42	177.06	199.87	46.83	55.21	4 020.33	3 910.67
Cardiology-Chat 8Bw/o RAG	90.77	102.37	157.11	178.64	27.84	33.28	4 331.24	4 210.88

**TABLE 5:** Calibration Performance (Brier Score and Expected Calibration Error)

Model	Brier		ECE	
	QA	In-house	QA	In-house
Qwen2.5-7B-Instruct	0.584	0.648	0.563	0.637
LLaMA-3.1-8B	0.631	0.694	0.617	0.688
BioMistral-7B	0.566	0.624	0.545	0.612
HuatuoGPT-o1-7B	0.553	0.606	0.532	0.598
R1-Distill-Llama-8B	0.536	0.584	0.547	0.572
Cardiology-Chat 8B	0.548	0.598	0.552	0.589
w/o RAG	0.612	0.685	0.601	0.671

#### 3) System-Level Efficiency and Retriever Quality

Cardiology-Chat 8B maintains efficient end-to-end runtime. QA: p50/p95 latency 105.5/177.1 ms, TTFT 46.8 ms, Throughput 4,020 tokens/s; in-house: 118.4/199.9 ms, 55.2 ms, 3,911 tokens/s. Compared with no-RAG, latency rises ~16% and throughput falls ~7%, still suitable for near-real-time use. The retriever shows high evidence quality on QA (Support@5 0.840, Relevant-Hit@5 0.878, Context Precision@5 0.587), supporting accurate, efficient reasoning.

### D. Ablation Study

To evaluate the independent contributions of the two key components—Reliable Chain-of-Thought (CoT) fine-tuning and the Structured Knowledge Base (KB) in the RAG framework—we conducted ablation experiments on both the QA benchmark and the in-house dataset (Tables 6–7).

**TABLE 6:** Ablation Study Results for Key Components of Cardiology-Chat (Accuracy)

Model	Structural Heart Disease	Functional Heart Disease	Coronary and Vascular Disease	Special Heart Diseases	Avg.
Baseline LLMs					
LLaMA-3.1-8B-Instruct	0.583	0.642	0.579	0.644	0.612
Effectiveness of reliable CoT					
SFT w/ simple CoT	0.601	0.693	0.591	0.663	0.637
SFT w/ reliable CoT	0.711	0.773	0.633	0.696	0.703
Effectiveness of Structured KB					
SFT w/o RAG	0.711	0.773	0.633	0.696	0.703
SFT+RAG w/o struct KB	0.731	0.756	0.694	0.726	0.727
SFT+RAG w struct(Ours)	0.807	0.851	0.76	0.766	0.796

**TABLE 7:** Ablation Study Results for Key Components of Cardiology-Chat (F1-Score and Qualitative Metrics)

Model	F1-score			LLM Qualitative Metrics		
	Precision	Recall	F1	Organization	Completeness	Succinctness
Baseline LLMs					
LLaMA-3.1-8B-Instruct	0.622	0.528	0.571	0.663	0.621	0.812
Effectiveness of reliable CoT					
SFT w/ simple CoT	0.647	0.593	0.619	0.692	0.661	0.820
SFT w/ reliable CoT	0.792	0.671	0.726	0.811	0.816	0.839
Effectiveness of struct KB					
SFT w/o RAG	0.792	0.671	0.726	0.811	0.816	0.839
SFT+RAG w/o struct KB	0.825	0.714	0.766	0.813	0.837	0.872
SFT+RAG w	0.853	0.766	0.807	0.827	0.874	0.787

**TABLE 8:** Retriever Performance Metrics

Metric	QA (n=182)	In-house (n=49)
Support@5	0.840	0.817
Relevant-Hit@5	0.878	0.801
Context Precision@5	0.587	0.632
Avg #Chunks	4.89	4.72
Avg Relevant	2.93	3.36
Avg Support	2.29	2.87
Avg Answer-Hit	2.46	3.21

Compared with the baseline LlaMA 3.1 8B-Instruct (average QA accuracy = 0.612, in-house F $1=0.571$), fine-tuning with simple CoT data already brings clear improvements (0.637 and 0.619, respectively). When replaced with reliable CoT, accuracy further rises to 0.703 on QA and 0.726 F1 in-house, demonstrating that enforcing logical and factual consistency in CoT sequences substantially enhances clinical reasoning capability.

Adding the RAG module with a structured KB provides additional performance gains. The structured KB version achieves 0.796 QA accuracy and 0.807 F1, compared with 0.703/0.726 (no RAG) and 0.727/0.766 (unstructured KB). These results indicate that well-structured, evidence-grounded retrieval supplies more relevant and coherent context, enabling the model to perform robustly across both benchmark and real-world diagnostic tasks. Overall, Reliable CoT and structured RAG act synergistically to strengthen factual grounding and reasoning completeness in Cardiology-Chat.

## V. Discussion

This study developed and evaluated Cardiology-Chat, a cardiology-specific diagnostic reasoning system that integrates a multi-LLM framework, a structured knowledge base, and Chain-of-Thought (CoT)-enhanced fine-tuning. The results across both QA benchmarks and in-house clinical data demonstrate substantial performance gains over baseline models. These improvements arise from the complementary strengths of the two key modules: the structured RAG component provides explicit evidence grounding and factual completeness, while reliable CoT fine-tuning strengthens logical coherence in multi-step reasoning. Ablation results confirm that removing either component markedly reduces diagnostic coverage and consistency. The slightly lower succinctness score (0.787 vs. 0.839 without RAG) reflects the expected trade-off of retrieval-augmented reasoning—enhanced completeness and interpretability at the cost of minimal verbosity, a desirable balance for clinical applications prioritizing safety and clarity.

Despite these promising results, certain methodological and practical limitations remain. The generation of CoT data currently relies on an automated dual-model validation loop (DeepSeek-V3 as generator and DeepSeek-R1 as validator) without direct clinician input. Although this heterogeneous setup effectively mitigates self-consistency bias and enforces factual accuracy, it cannot yet capture nuanced clinical reasoning in ambiguous or multi-morbidity cases. Future work will involve iterative refinement with expert-reviewed CoT data to ensure medical validity and transparency.

Preliminary inspection of in-house predictions suggests that most failure cases occurred in coronary artery disease and heart failure cohorts, where overlapping symptoms and comorbidities complicate differential diagnosis. Typical errors include missing secondary diagnoses or confusion between ischemic and non-ischemic etiologies. Expanding the in-house dataset will allow a more systematic error taxonomy, helping to identify reasoning gaps and guide targeted retraining of domain-specific reasoning patterns.

Finally, while the LLM-based qualitative evaluation offers a scalable proxy for expert review, it lacks clinical nuance and may introduce evaluator bias. Hence, these qualitative metrics should be viewed as indicative rather than definitive. Moving forward, large-scale, multi-center validation, continuous knowledge-base updating, and integration of multimodal clinical data (e.g., ECG, echocardiography) will be essential to transition Cardiology-Chat from experimental validation to practical clinical decision support. Despite current limitations, the proposed architecture provides a robust and interpretable paradigm for domain-specialized medical reasoning, bridging the gap between general LLM capabilities and real-world cardiology applications.

## VI. Conclusion

This study successfully developed and validated Cardiology-Chat, which addresses complex challenges in cardiology diagnosis by integrating a multi-LLM-supported three-step reasoning framework, a specialized knowledge base, and a CoT enhancement strategy. The system innovatively combines RAG with SFT, leveraging a task-optimized multi-LLM approach. Specifically, fine-tuning Llama 3.1 8B-instruct with reliable CoT data—generated using distinct DeepSeek models (V3 for generation, R1 for validation)—enhances specialized knowledge acquisition and complex clinical reasoning. Experimental results demonstrate that Cardiology-Chat significantly outperforms general LLM baselines on cardiology multiple-choice question benchmarks and real clinical case diagnostic tasks, demonstrating excellent performance. Ablation studies confirm the critical contributions of the structured knowledge base and reliable CoT fine-tuning within this tailored multi-LLM architecture. This investigation validates a robust paradigm for combining domain knowledge retrieval with deep reasoning, offering empirical support and a developmental roadmap for reliable, specialized medical LLMs. However, challenges remain, including the need for broader generalizability across diverse patient populations and the integration of real-time clinical feedback to refine model outputs. Future work should explore incorporating multi-modal data, such as echocardiograms, to enhance diagnostic precision and develop mechanisms for continuous knowledge base updates to reflect evolving medical guidelines, further strengthening clinical applicability.

## References

[1] E. Board, “Cardiovascular risk in the workplace,” Eur. J. Occupational Health Nursing, vol. 3, no. 2, pp. 1–3, Dec. 2024, doi: 10.70324/ejohn.v4i2.41.

[2] H. Sadr, A. Salari, M. T. Ashoobi, and M. Nazari, “Cardiovascular disease diagnosis: A holistic approach using the integration of machine learning and deep learning models,” Eur. J. Med. Res., vol. 29, no. 1, p. 455, Sep. 2024. 39261891 10.1186/s40001-024-02044-7 PMC11389500

[3] A. Rahman , “Comparative analysis based on DeepSeek, ChatGPT, and Google gemini: Features, techniques, performance, future prospects,” 2025, arXiv:2503.04783.

[4] G. Quer and E. J. Topol, “The potential for large language models to transform cardiovascular medicine,” Lancet Digit. Health, vol. 6, no. 10, pp. e767–e771, Oct. 2024. 39214760 10.1016/S2589-7500(24)00151-1

[5] Z. A. Nazi and W. Peng, “Large language models in healthcare and medical domain: A review,” in Proc. Informat., 2024, p. 57.

[6] A. Sarraju, D. Ouyang, and D. Itchhaporia, “The opportunities and challenges of large language models in cardiology,” JACC, Adv., vol. 2, no. 7, Sep. 2023, Art. no. 100438. 10.1016/j.jacadv.2023.100438 PMC11198078 38939505

[7] R. Hou , “MSDiagnosis: An EMR-based dataset for clinical multi-step diagnosis,” 2024, arXiv:2408.10039.

[8] K. Singhal , “Large language models encode clinical knowledge,” Nature, vol. 620, no. 7972, pp. 172–180, 2023. 37438534 10.1038/s41586-023-06291-2 PMC10396962

[9] K. K. Y. Ng, I. Matsuba, and P. C. Zhang, “RAG in health care: A novel framework for improving communication and decision-making by addressing LLM limitations,” NEJM AI, vol. 2, no. 1, Jan. 2025, Art. no. 2400380.

[10] X. Xu , “A comprehensive review on synergy of multi-modal data and AI technologies in medical diagnosis,” Bioengineering, vol. 11, no. 3, p. 219, Feb. 2024. 38534493 10.3390/bioengineering11030219 PMC10967767

[11] A. Bonfigli, L. Bacco, M. Merone, and F. Dell’Orletta, “From pre-training to fine-tuning: An in-depth analysis of large language models in the biomedical domain,” Artif. Intell. Med., vol. 157, Nov. 2024, Art. no. 103003. 10.1016/j.artmed.2024.103003 39471773

[12] P. Lewis , “Retrieval-augmented generation for knowledge-intensive NLP tasks,” in Proc. Adv. Neural Inf. Process. Syst., vol. 33, 2020, pp. 9459–9474.

[13] S. Borgeaud , “Improving language models by retrieving from trillions of tokens,” in Proc. Int. Conf. Mach. Learn., 2021, pp. 2206–2240.

[14] G. Frisoni, M. Mizutani, G. Moro, and L. Valgimigli, “Bioreader: A retrieval-enhanced text-to-text transformer for biomedical literature,” in Proc. Conf. Empirical Methods Natural Lang. Process., 2022, pp. 5770–5793.

[15] H. Ahsan , “Retrieving evidence from EHRs with LLMs: Possibilities and challenges,” in Proc. Mach. Learn. Res., vol. 248, 2023, pp. 489–505. PMC11368037 39224857

[16] R. M. Wehbe , “Deep learning for cardiovascular imaging: A review,” JAMA Cardiol., vol. 8, no. 11, pp. 1089–1098, 2023. 37728933 10.1001/jamacardio.2023.3142

[17] F. Syryca, C. Gräßer, T. Trenkwalder, and P. Nicol, “Automated generation of echocardiography reports using artificial intelligence: A novel approach to streamlining cardiovascular diagnostics,” Int. J. Cardiovascular Imag., vol. 41, no. 5, pp. 967–977, Mar. 2025. 10.1007/s10554-025-03382-1 PMC12075404 40159559

[18] M. Ahmed, J. Lam, A. Chow, and C.-M. Chow, “A primer on large language models (LLMs) and ChatGPT for cardiovascular healthcare professionals,” CJC Open, vol. 7, no. 5, pp. 660–666, May 2025. 40433202 10.1016/j.cjco.2025.02.012 PMC12105510

[19] Y. Zhou , “Zodiac: A cardiologist-level LLM framework for multi-agent diagnostics,” 2024, arXiv:2410.02026.

[20] Y.-L. Li , “Predicting long-term time to cardiovascular incidents using myocardial perfusion imaging and deep convolutional neural networks,” Sci. Rep., vol. 14, no. 1, p. 3802, Feb. 2024. 38360974 10.1038/s41598-024-54139-0 PMC10869727

[21] H. Yu, P. Guo, and A. Sano, “Zero-shot ECG diagnosis with large language models and retrieval-augmented generation,” in Proc. Mach. Learn. Health, 2023, pp. 650–663.

[22] T. Tran , “CardioCanon: A customised chatbot for cardiology inquiry with retrieval augmented generation to reduce hallucinations and improve performance of large language models,” Heart, Lung Circulat., vol. 33, pp. S379–S380, Aug. 2024.

[23] J. Tang, T. Xia, Y. Lu, C. Mascolo, and A. Saeed, “Electrocardiogram report generation and question answering via retrieval-augmented self-supervised modeling,” 2024, arXiv:2409.08788.

[24] J. Wei , “Chain-of-thought prompting elicits reasoning in large language models,” in Proc. Adv. Neural Inf. Process. Syst., vol. 35, 2022, pp. 24824–24837.

[25] X. Wang , “Self-consistency improves chain of thought reasoning in language models,” 2022, arXiv:2203.11171.

[26] Y. Song, T. Wang, P. Cai, S. K. Mondal, and J. P. Sahoo, “A comprehensive survey of few-shot learning: Evolution, applications, challenges, and opportunities,” ACM Comput. Surv., vol. 55, no. 13s, pp. 1–40, Dec. 2023.

[27] J. Chen , “HuatuoGPT-o1, towards medical complex reasoning with LLMs,” 2024, arXiv:2412.18925.

[28] A. Grattafiori , “The llama 3 herd of models,” 2024, arXiv:2407.21783.

[29] D. Jin, E. Pan, N. Oufattole, W.-H. Weng, H. Fang, and P. Szolovits, “What disease does this patient have? A large-scale open domain question answering dataset from medical exams,” Appl. Sci., vol. 11, no. 14, p. 6421, Jul. 2021.

[30] A. Pal, L. K. Umapathi, and M. Sankarasubbu, “MedMCQA: A large-scale multi-subject multi-choice dataset for medical domain question answering,” in Proc. Conf. Health, Inference, Learn., 2022, pp. 248–260.

[31] A. Liu , “DeepSeek-V3 technical report,” 2024, arXiv:2412.19437.

[32] D. Guo , “DeepSeek-r1: Incentivizing reasoning capability in LLMs via reinforcement learning,” 2025, arXiv:2501.12948.

[33] Q. Jin, B. Dhingra, Z. Liu, W. W. Cohen, and X. Lu, “PubMedQA: A dataset for biomedical research question answering,” 2019, arXiv:1909.06146.

[34] Qwen2.5: A Party of Foundation Models, Qwen Team, Alibaba Group, Hangzhou, China, Sep. 2024.

